# Supplementary material for: The type VI secretion system sheath assembles at the end distal from the membrane anchor
Source: Nat Commun. 2017 Jul 13;8:16088. doi: 10.1038/ncomms16088 (PMC5511345; doi:10.1038/ncomms16088)
Supplement: Supplementary Information [file ncomms16088-s1.pdf]

File name: Supplementary Information
Description: Supplementary Figures and Supplementary Table

File name: Supplementary Movie 1
Description: T6SS remains dynamic during spheroplast formation. VipA-msfGFP and ClpVmCherry2 labeled V. cholerae cells were grown to OD = 0.8 and imaged on a 1 % agarose LB pad containing 500 µg ml^{-1} ampicillin. Five representative 90 min time lapse series acquired with a rate of 1 min $frame^{-1}$ for each time point are shown. Field of view is 20 x 20 µm and shows a merge of phase contrast and two fluorescent channels: VipA-msfGFP and ClpV-mCherry2. Furthermore, GFP and mCherry2 fluorescence channels are depicted individually on the right as grey scale images. The video plays at a rate of 10 frames per second.

File name: Supplementary Movie 2
Description: T6SS dynamics in rod shaped cells are comparable to spheroplasts. Spheroplast were induced by the addition of 500 µg ml^{-1} ampicillin for indicated time points. Cells were imaged on a 1% agarose LB pad. Three representative 5 min time lapse series acquired with a rate of 10 sec $frame^{-1}$ for each time-point are shown. Field of view is 5 x 5 µm and shows a merge of phase contrast and three fluorescent channels: VipA-msfGFP, ClpV-mCherry2 and HADA labeling of PG. Furthermore, GFP and mCherry2 fluorescence channels are depicted individually on the right as grey scale images. The video plays at a rate of 10 frames per second.

File name: Supplementary Movie 3
Description: T6SS dynamics in ampicillin induced V. cholerae spheroplasts. Spheroplasts were induced by the addition of 500 µg ml^{-1} ampicillin for 40 min and subsequently imaged on a 1 % agarose LB pad. Three representative 5 min time lapse series acquired with a rate of 10 sec $frame^{-1}$ are shown. Field of view is 50 x 50 µm and shows a merge of phase contrast and three fluorescent channels: VipA-msfGFP, ClpV-mCherry2 and HADA labeling of PG. The video plays at a rate of 10 frames per second.

File name: Supplementary Movie 4
Description: T6SS in spheroplasts is capable of translocating VgrG2 into cytosol of recipient cells. Recipient ΔvgrG2/vipA-msfGFP spheroplasts were co-incubated with T6SS+ clpVmCherry2 donor cells on a 1 % agarose LB pad. The sheath assembly was monitored in GFP channel. Five representative time-lapse image series acquired for 5 min with a rate of 10 sec $frame^{-1}$ are shown. Field of view is 10 x 10 µm and shows a merge between bright-field, mCherry2 and GFP fluorescence channels on the left; right field shows only GFP fluorescence channel. The video plays at a rate of 7 frames per second.

File name: Supplementary Movie 5
Description: T6SS in spheroplasts is capable of killing target cells. VipA-msfGFP labeled spheroplasts were co-incubated with MG1655 prey cells on agarose pads containing 100 µg ml^{-1} ampicillin and 1 µg ml^{-1} propidium iodide. Cell death was identified by detection of propidium iodide staining. Five representative time-lapse image series acquired for 45 min with a rate of 30 sec $frame^{-1}$ are shown. Field of view is 10 x 10 µm and shows a merge between bright-field, propidium iodide and GFP fluorescence channels. The video plays at a rate of 10 frames per second.

File name: Supplementary Movie 6
Description: New sheath subunits are incorporated at the end distal from the assembly initiation. VipA-msfGFP labeled ΔvgrG1/ΔvasX spheroplasts (ampicillin 500 µg ml^{-1}, 40 min) were monitored for sheath assembly for 2 min at a rate of 2 sec $frame^{-1}$. After 30 sec of image acquisition, the bleaching

laser was triggered. Ten representative time-lapse series are shown. Field of view is 5 x 5 µm. The video plays at a rate of 10 frames per second.

File name: Peer Review File
Description:

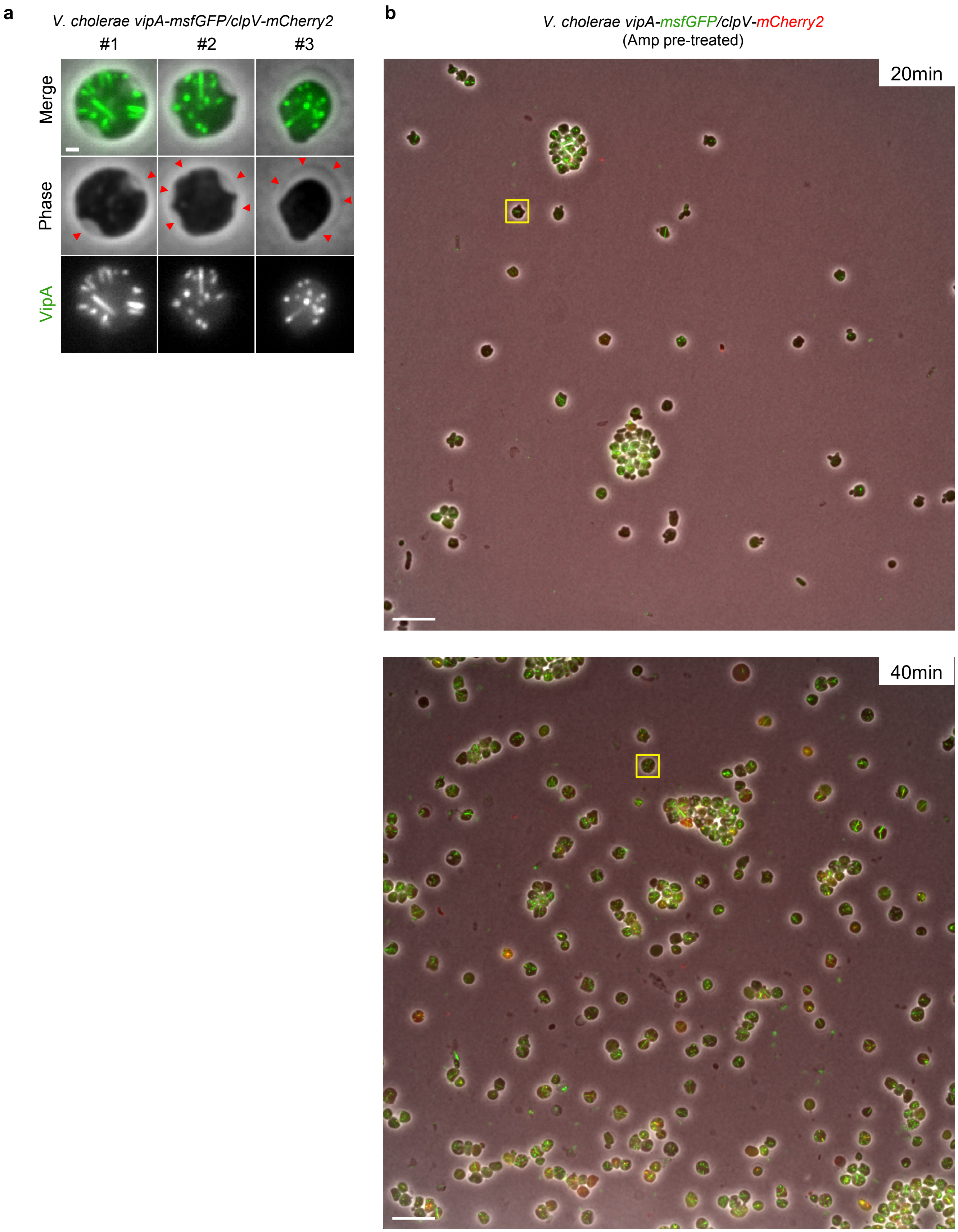

Supplementary Figure 1: T6SS dynamics in spheroplasts. (a) Examples of membrane detachment (arrow heads) in spheroplasts (*vipA-msfGFP* background) that were incubated for 60 min in the presence of ampicillin (500 µg ml^{-1}). Scale bar = 1 µm. **(b)** Full (133 x 133 µm) fields of view are shown for cells that were incubated either for 20 min (top) or 40 min (bottom) in the presence of ampicillin. Yellow boxes mark the cropped regions shown in Figure 1a. Scale bar = 10 µm.

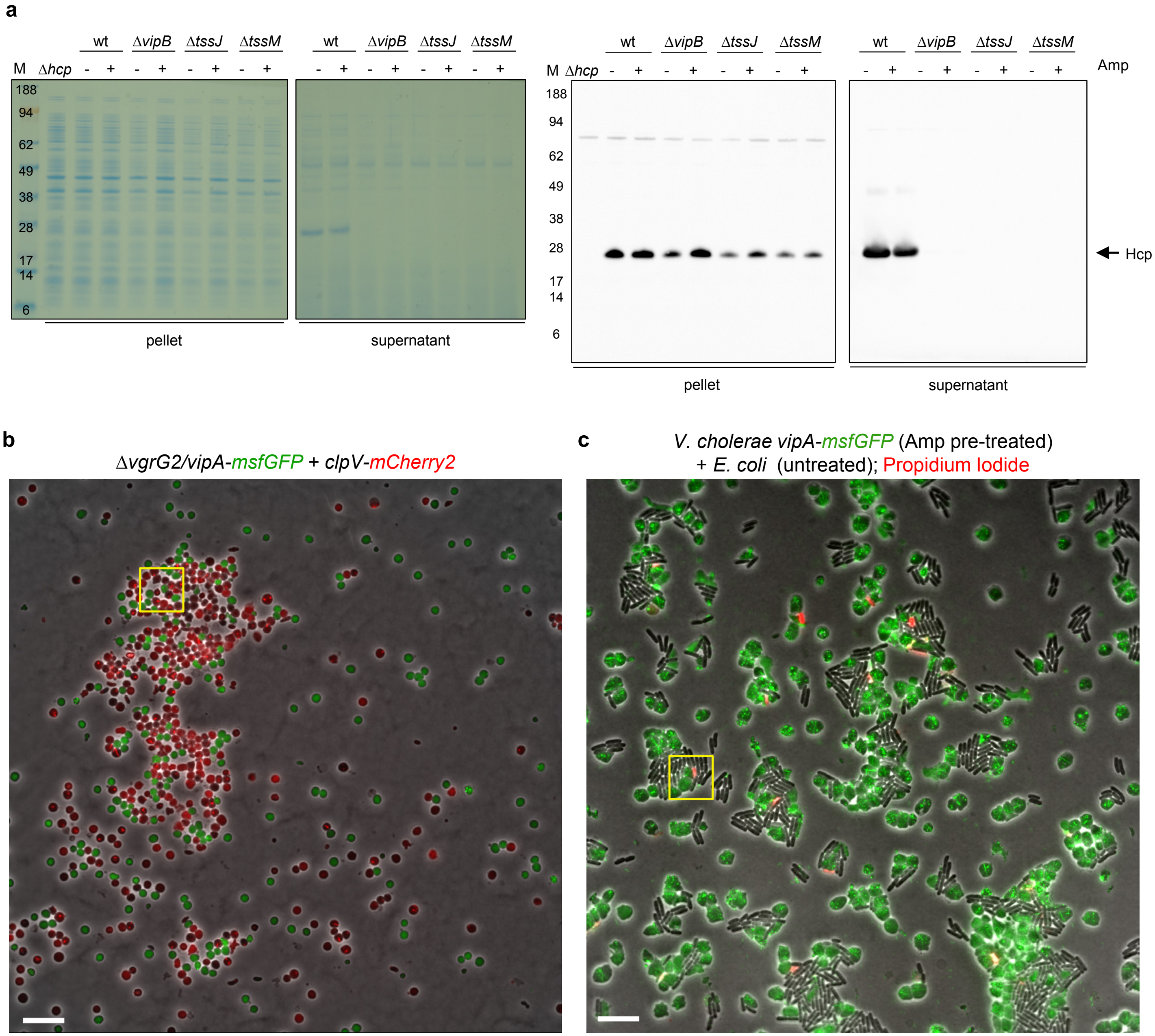

Supplementary Figure 2: The T6SS apparently remains functional in ampicillin-induced spheroplasts. (a) Cell lysates and precipitated culture supernatants of spheroplasts and rod shaped cells of indicated strains were separated by SDS-PAGE and stained with Coomassie Blue (left) or probed with polyclonal antibody raised against recombinant full-length Hcp (right). Arrows point towards specific bands for Hcp (theoretical MW = 19.1 kDa). Marker for molecular weight is indicated on the right in kDa. **(b-c)** Full (133 x 133 µm) fields of view are shown for VgrG2 interbacterial protein complementation assay **(b)** and *E. coli* cell permeabilization assay **(c)**. Yellow boxes mark cropped regions shown in Figure 2b and d respectively. Scale bar = 10 µm

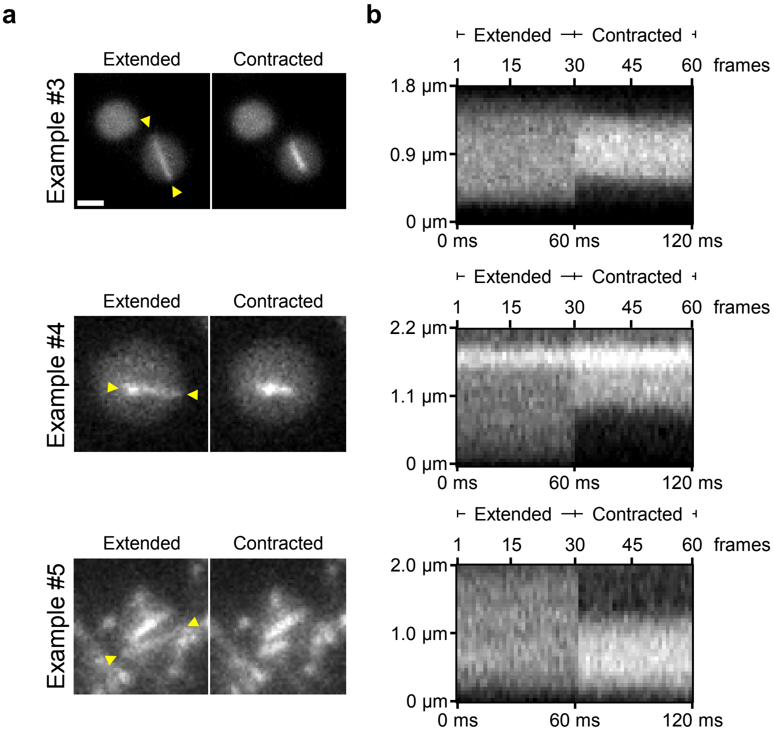

c

N	Sheath length [µm]		Ext - Cont	Contration [%]
	Extended	Contracted		
1	1.55	0.79	0.76	**49.03**
2	1.66	0.89	0.77	**46.39**
3	2.14	1.01	1.13	**52.80**
4	1.91	1.12	0.79	**41.36**
5	3.29	1.69	1.6	**48.63**

Supplementary Figure 3: Additional examples of sheath contractions. (a) Additional examples of sheath contraction acquired at a rate of 500 frames sec^{-1} and corresponding **(b)** kymograms are depicted as shown in Figure 3. **(c)** Corresponding measurements to determine level of contraction. Sheath length from the last frame prior to contraction and the first frame after contraction were measured. Calculated length difference was normalized to the extended sheath length.

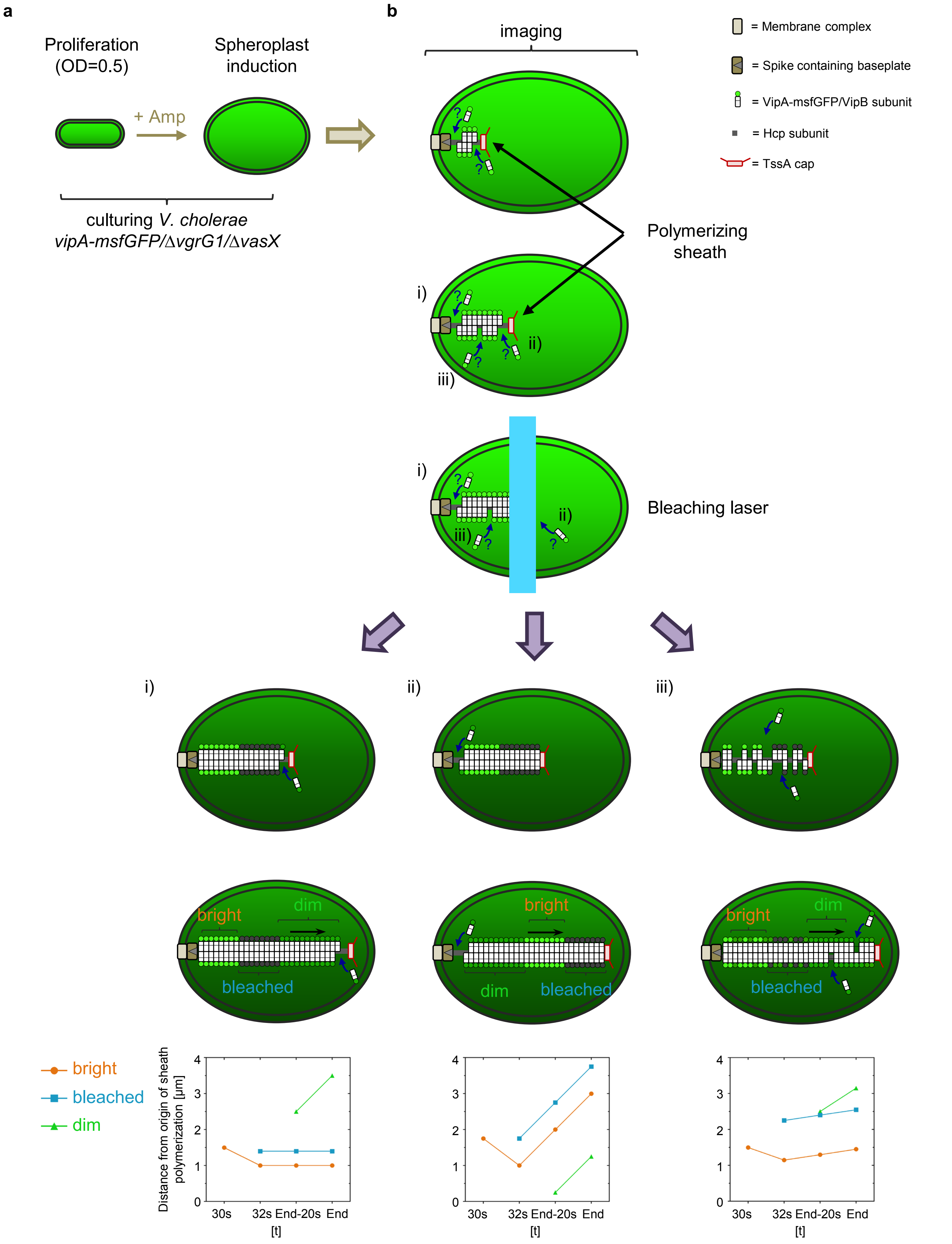

Stationary bright and bleached sections near the baseplate

Bright and bleached sections **move** from the baseplate.

Slow moving bleached section with **fluorescence recovery**.

Supplementary Figure 4: Experimental approach to determine site of sheath subunit incorporation. (a) VipA-msfGFP labeled *V. cholerae* lacking *vgrG1* and *vasX* were grown to an OD = 0.5 and exposed to 500 µg ml^{-1} ampicillin to induce spheroplast formation. **(b)** Three models were tested by monitoring sheath polymerization after photobleaching: (i) incorporation of new subunits at the distal cytosolic end, (ii) at the baseplate and (iii) random incorporation along the entire polymer. After photobleaching, 3 signal intensities should be identified: (i) the ”bright” section consisting of assembled sheath subunits prior to bleaching; (ii) the “bleached” section representing the part of the sheath, which was directly hit by the bleaching laser and (iii) the ”dim” section consisting of partially bleached sheath subunits present in the cytosol. Based on the localization and fluorescence intensity changes of these sections in time, the three proposed models for sheath assembly can be distinguished.

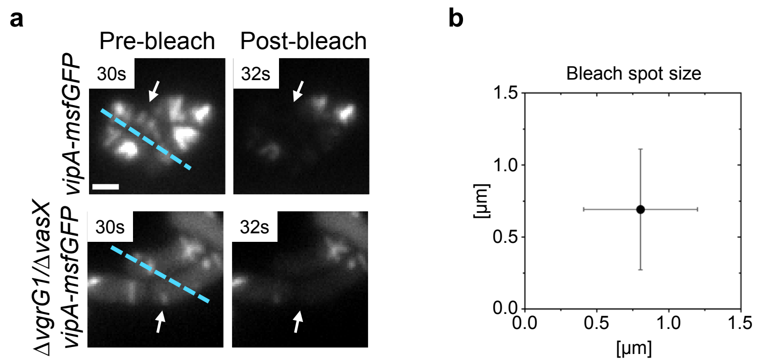

Supplementary Figure 5: Photobleaching experiments are impossible to perform on rod shaped cells. (a) Polymerizing sheaths (arrows) of indicated strains were imaged for 2 min and photobleached (λ = 488 nm; 100 % laser power; 0.1 ms pixel^{-1}) after 30 sec. The last frame prior and the first frame after photobleaching are depicted. **(b)** The bleach spot size was measured in spheroplasts from 25 successfully photobleached sheaths. Data are represented as mean +/- SD. Scale bar = 1 µm.

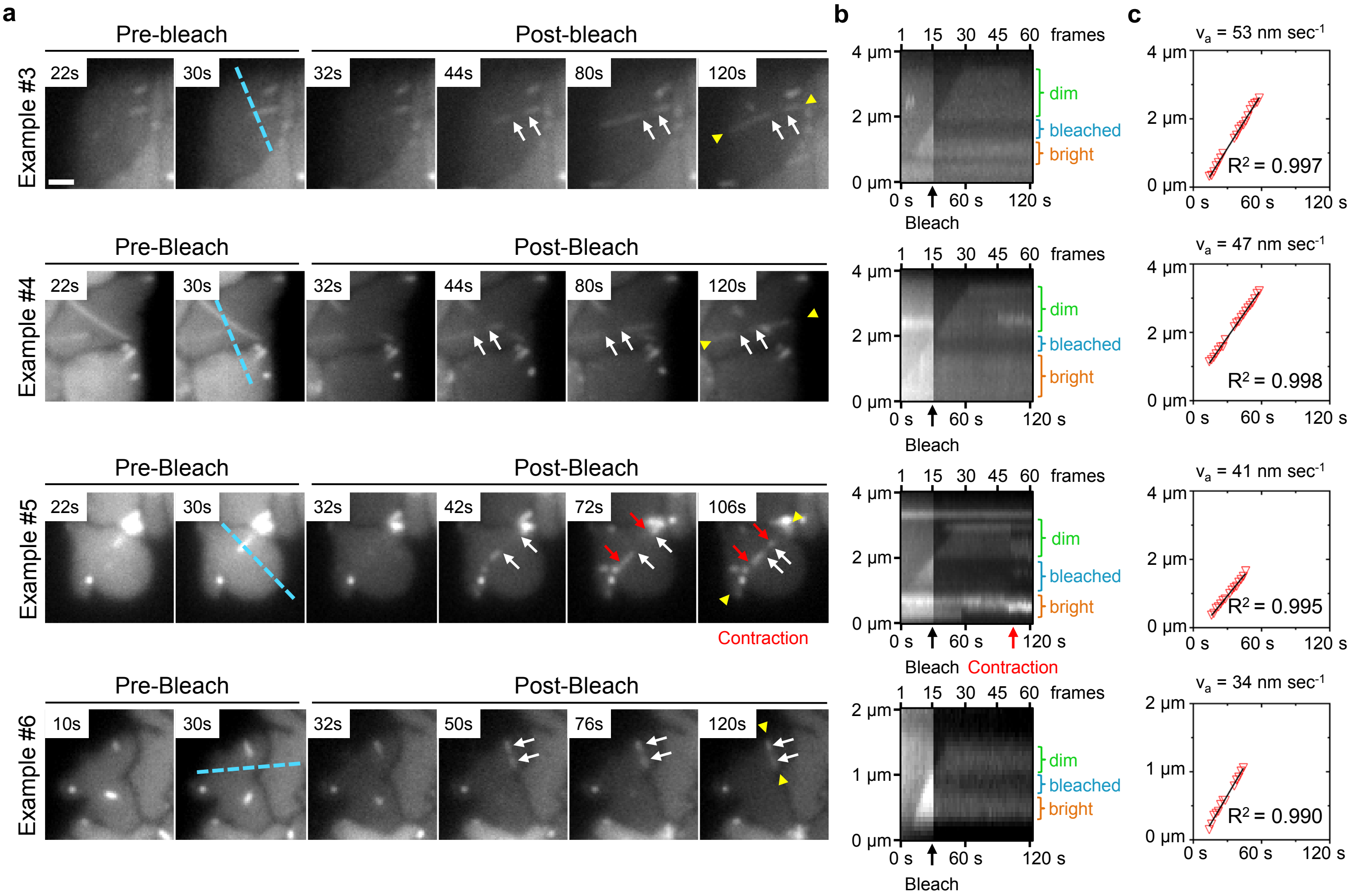

Supplementary Figure 6: Photobleached sections of sheaths remain stationary and with unchanged intensity after photobleaching. (a) Additional examples of successfully photobleached polymerizing sheaths as well as corresponding **(b)** kymograms and polymerization speed measurements **(c)** as shown in Figure 5. Red arrows indicated sheath contraction of a photo bleached polymer.

Supplementary Table 1: Strains used in this study, related to Methods

Organism	Genotype	Plasmid	Relevant features	Source
V. cholerae 2740-80	lacZ$^-$, Str^r		Parental strain	(Basler et al., 2012)
	lacZ$^-$, Str^r	pBAD24	Parental strain, Amp^r	This study
	lacZ$^-$, Str^r, Δ*vipB*		Deletion of *vipB*; T6SS negative	(Basler et al., 2012)
	lacZ$^-$, Str^r, Δ*vipB, vipA-msfGFP*		Deletion of *vipB*; T6SS negative in *vipA-msfGFP* background	(Kudryashev et al., 2015)
	lacZ$^-$, Str^r, Δ*tssJ, vipA-msfGFP*		Deletion of outer membrane protein *tssJ* in *vipA-msfGFP* background	This study
	lacZ$^-$, Str^r, Δ*tssM, vipA-msfGFP*		Deletion of membrane protein *tssM* in *vipA-msfGFP* background	This study
	lacZ$^-$, Str^r, *vipA-msfGFP, clpV-mCherry2*		C-terminal chromosomal fusions of *msfGFP* to *vipA* and *mCherry2* to *clpV*	(Basler and Mekalanos, 2012)
	lacZ$^-$, Str^r, *clpV-mCherry2*		C-terminal chromosomal fusion of *mCherry2* to *clpV*	(Basler and Mekalanos, 2012)
	lacZ$^-$, Str^r, *vipA-msfGFP*		C-terminal chromosomal fusion of *msfGFP* to *vipA*	(Kudryashev et al., 2015)
	lacZ$^-$, Str^r, *vipA-msfGFP,* Δ*vipB*		Deletion of *vipA*; T6SS negative	(Kudryashev et al., 2015)
	lacZ$^-$, Str^r, *vipA-msfGFP,* Δ*vgrG2*		Deletion of *vgrG2* in *vipA-msfGFP* background	(Vettiger and Basler, 2016)
	lacZ$^-$, Str^r, *vipA-msfGFP,* Δ*vgrG1,* Δ*vasX*		Combination of *vgrG1* and *vasX* deletions in *vipA-msfGFP* background	(Vettiger and Basler, 2016)
E. coli MG1655	K-12, F$^-$, λ$^-$, *ilvG*$^-$, *rfb-50, rph-1, lacZ+*	pUC19	Used for quantitative killing assays, Amp^r	(Kudryashev et al., 2015)
